# Prospecting Stress Formed by Hydrogen or Isotope Diffused in Palladium Alloy Cathode

**DOI:** 10.3390/ma11112101

**Published:** 2018-10-26

**Authors:** Gilbert Bellanger

**Affiliations:** Independent Corrosion Expert; Selongey 21260, France; gilbert.bellanger@orange.fr; Tel.: +33(0)-380-757-610

**Keywords:** hydrogen, tritium, electrolyzer, cathode, diffusion, palladium alloy, stress corrosion cracking

## Abstract

The objective of this project is to take into account the mechanical constraints formed by diffusion of hydrogen or tritium in watertight palladium alloy cathode. To know the origin of these, it was necessary to discriminating the damaging effects encountered. Effectively, hydrogen and isotope induce deformation, embrittlement, stress corrosion cracking and cathodic corrosion in different regions of cathode. Palladium can be alloyed with silver or yttrium to favourably increase diffusion and reduce these constraints. Effects of electrochemical factors, temperature, cathode structure, adsorbed transient complex of palladium and porous material support are given to estimate and to limit possible damage.

## 1. Introduction to the Electrolysis Model

**Figure 1:**
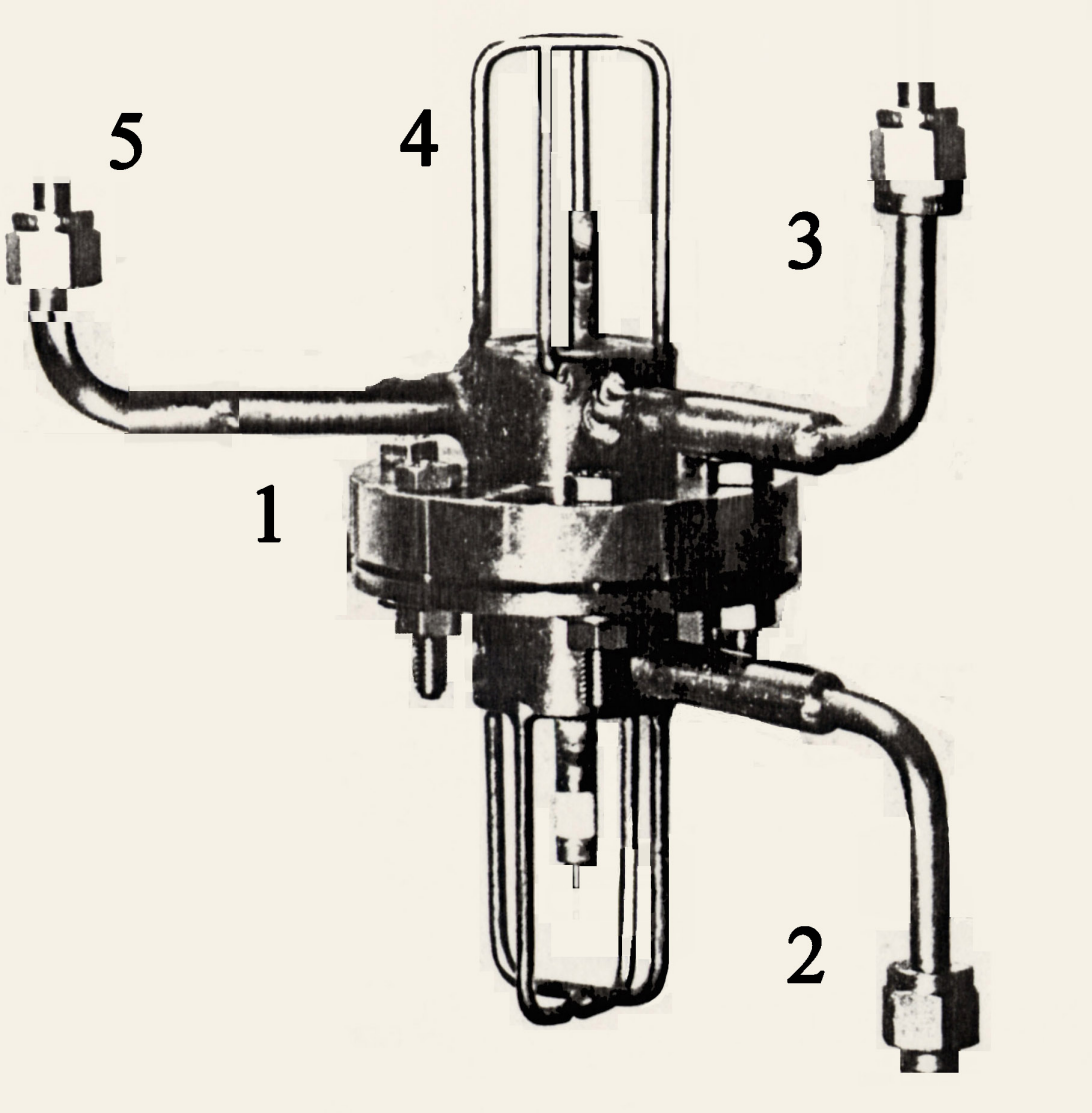
Device working with flat diffusion cathode (1) electrolyzer, (2) towards tank for hydrogen storage, (3) oxygen extraction, (4) water injection, (5) neutral gas sweeping.

Hydrogen is an alternative fuel more efficient than oil or gas. It meets energy needs in reducing dependence on fossil fuels. When it is produced from the electrolysis of water and if electricity used is produced from renewable sources, it is an inexhaustible resource that respects the environment with zero-emission of greenhouse gases. One attractive way of producing high purity hydrogen is electrolysis using palladium diffusion cathode at double face [1]. In another application concerning nuclear fusion energy systems, tritiated water can be treated by this innovating electrolysis process using the same palladium cathode after isotopic enrichment [2]. Limiting the tritiated water stock for long duration in nuclear containers helps to reduce the risk of localized corrosion impacting environment by contamination [3]. In these processes, stress corrosion cracking depends on diffusing hydrogen or isotope and structure of metal. Concept and scheme of palladium cathode shaped as a hollow glove finger or a flat disk are given in references [4,5]. Electrolyzer can be combined with a chromatographic systems to separate hydrogen isotopes [6]. The opened end of glove finger is connected to the tank for storage of pure hydrogen and isotope [1]. In reference [5], the electrolyzer concept is the easy inter-changeability of flat cathode (Figure 1). Each hydrogen isotope has the same stress cracking mechanism for the palladium cathode. Difference concerns the respective size of diffusing atom or ion, values of diffusion in palladium and of flow in porous material used as support inside the hollow finger. There is also the positive charge helium formed from the decaying tritium, the environmental nuisance due to radioactivity, thereby the difficulty to work in confined enclosure for safety. Considering energy of the $\beta^{-}$ ray formed from decaying, this is not strong enough to break metal bonds, but on the other hand sufficient to break oxide bonds protecting stainless steel, oxide breaking is responsible of localized corrosion where corrosion resistance is required [7]. The problem addressed in this paper is not diffusion, but embrittlement and cracking mechanisms, therefore we have written the hydrogen without distinction of isotope for simplicity. This cracking mechanism is incomparably much more intense than that for the gas-gas diffusion in palladium alloy membranes. Therefore this problem should not be underestimated, and electrolytic embrittlement requires that it must be brought under control before carrying out the industrial electrolytic process. Electrolyzer is completely automatic and is equipped with diffused hydrogen pressure controller, in-process water calculator and temperature regulator [1]. Oxygen generated at the anode during electrolysis escapes through an outlet in the top of electrolyzer.

Stainless steel used for electrolyzer is inert to alkaline corrosion [8]. Permeability (*P*) in palladium cathode depends on the activation energy whereas flow in porous material used as support is a direct function of size of isotope. In the palladium cathode, hydrogen is adsorbed as a proton on the electrode surface and diffuses into the metal according to Fick’s equations. Protons permeated through the thickness of cathode are desorbed as pure molecular hydrogen before flowing in the porous support, or directly re-oxidized on the output side of electrode for isotopic enrichment. A mathematical treatment for the first Fick’s equation applicable to hydrogen in palladium has been given in [9,10]. Hydrogen dissolves with two non-stoichiometric phases and with concentration discontinuity between them. It is shown how the discontinuity modifies the global diffusion coefficient, permeability and the time-lag response in the transitory time. Equation can be summarized in Equation (1).

(1) $$P_{\alpha ,\beta }=-\sum \left[{\left[D\frac{\partial C}{\partial x}\right]}_{\alpha },{\left[D\frac{\partial C}{\partial x}\right]}_{\beta }\right]$$

where *P* is the permeability, **D** is the diffusion coefficient. In Equation (1), permeability is depending on each diffusion coefficient and the derivative of hydride solubility (*∂*C) for a defined thickness (*∂*x) into each phase. This system at multiphase could lead to stress into cathode if steady-state equilibrium for the two phases is not reached during diffusion. Hydride promotes permeation but on other hand can facilitate stress corrosion cracking [11]. In final, dislocations in metal would act as hydrogen traps leading to decrease performance. At ambient temperature, hydrogen diffuses too few in the palladium cathode and higher temperature is selected for industrial process (Equations (2)–(4)). For that, electrolyte can be NaOH concentrated at 18 mol dm${}^{-3}$ or a mixture KOH 50 mol dm${}^{-3}$ and LiOH 17 mol dm${}^{-3}$ or a pressure of 10${}^{3}$ kPa in the electrolyzer for temperature over 180 ${}^{{}^{\circ}}$C until 250 ${}^{{}^{\circ}}$C [12]. Unfortunately, using these concentrated electrolytes or high pressure with high temperature causes serious problem of safety and, their use is not recommended for the industrial electrolyzer. Electrolysis and diffusion induce two inverted isotopic effects depending on charging and diffusion [4].

## 2. Initial Observations and Formulation of the Problem

It is unthinkable to make without concerted approach an industrial electrolyzer using a diffusion cathode from usual metals as iron or nickel for economic purpose. Making it, severe structural deformations with consequent hysteretic behaviors are remarked, and diffusion results will be outside those expected. Consequently, this industrial electrolyzer will be useless, of expensive cost and of a large loss of time. The first thing to do is to interrogate the behavior for corrosion-assisted fracture and values of permeability available in the literature. For such metals, fractures are produced and permeability values are really too small. In brief, to avoid all inconvenient, the project has to be realistic to achieve the expected results. After this clarification, the following discussion describes optimization of the palladium-based cathode. Before examining the palladium alloys, pure palladium is an ideal material for hydrogen charging, but it has unsatisfactory mechanical property for hydrogen diffusion. Effectively, the metal lattice expands by formation of three immiscible hydride phases producing rupture of finger or disk by stress corrosion with transgranular cracks formation for lower critical temperature than 295 ${}^{{}^{\circ}}$C [11]. These are the brittle hydride β-phase expanding the lattice in the inlet side, the crossing α+β-phase of intermediate miscibility and the ductile α-phase in the outlet side. In the inlet side, conversion of α-phase, poor in hydrogen, into β-phase, rich in hydrogen, corresponds to an irreversible volume increase of 10%, and will result in different lattice spacing, high internal stress and cathode warping. The stresses cause a decrease in the life of the cathode, therefore the design must minimize the deformation [13]. High permeability (cm${}^{3}$ cm${}^{-2}$ s${}^{-1}$) is expected by the relative contribution of diffusion coefficient, large hydrogen concentration gradient (Δ**C**) between the inlet and outlet sides and small palladium cathode thickness (**e**) expressed by the simplified Equations (2)–(4):

(2) $$P=\left[\frac{D\Delta C}{e}\right]$$

where the concentration gradient is (Equation (3)):

(3) $$\Delta C=k\sqrt{2}\exp\left[\frac{(1-\alpha )\eta F}{2RT}\right]$$

where the diffusion coefficient is given by Equation (4):

(4) $$D=D_{0}\exp\left[\frac{W}{\mathrm{RT}}\right]$$

**k** is a constant, α the coefficient for electrical charge, η the cathodic overpotential related to normal hydrogen electrode, **F** Faraday constant, **T** the absolute temperature, **W** is the activation energy, **D**${}_{0}$ is a diffusion constant whose numerical value is that for **T**${}^{-1}$ is zero and **R** the gas constant. The minimum thickness below which permeation becomes free is a few μm. Palladium cathode at very small thickness is not able to resist at undesirable indirect effects due to temperature deviations and mechanical constraints, and suffers embrittlement by hydrogen [14]. It is the same problem for a thin cathode in palladium obtained by sputtering, and these have not been accepted for industrial application [15]. Such thin waterproof cathode would not be able to withstand a large surface without deformation for this industry which must be safe. In this context, unfortunately a ductile fracture would occur. Since the palladium cathode has not the mechanical quality, it requires porous support that has to contain lattice expansions across the finger or disk for temperature over the critical temperature [16]. Hydrogen or isotope flow across support is depending on porosity. Pores have to be tiny little to avoid local crushing of cathode at the aperture of these. As the temperature increases, the rate of proton diffusion through the cathode increases whereas the molecular hydrogen flow through the support decreases [17]. Therefore, support will be able to govern diffusion in the cathode. If the size of pore or their volume fraction is too small, or if thickness of support is too large, a fraction of hydrogen will be stopped at the palladium-support interface. The system is no more under steady state condition. The blocked hydrogen will back diffuse until the electrolyte or will inflate the palladium at the cathode-support interface by inner pressure formation leading to stress corrosion cracking. Depending on characteristics of porous support, pores can offer considerable resistance to flux. Therefore support constitutes the third barrier to cross for the process, and electrolysis and diffusion are the two others. Moreover, porous support induces the third isotopic effect depending on the size of hydrogen isotope molecule. Discharge, diffusion and flowing have to be equilibrated from the slowest process to give the global isotopic effect. If not, the slow process will alone govern the global isotopic effect.

**Figure 2:**
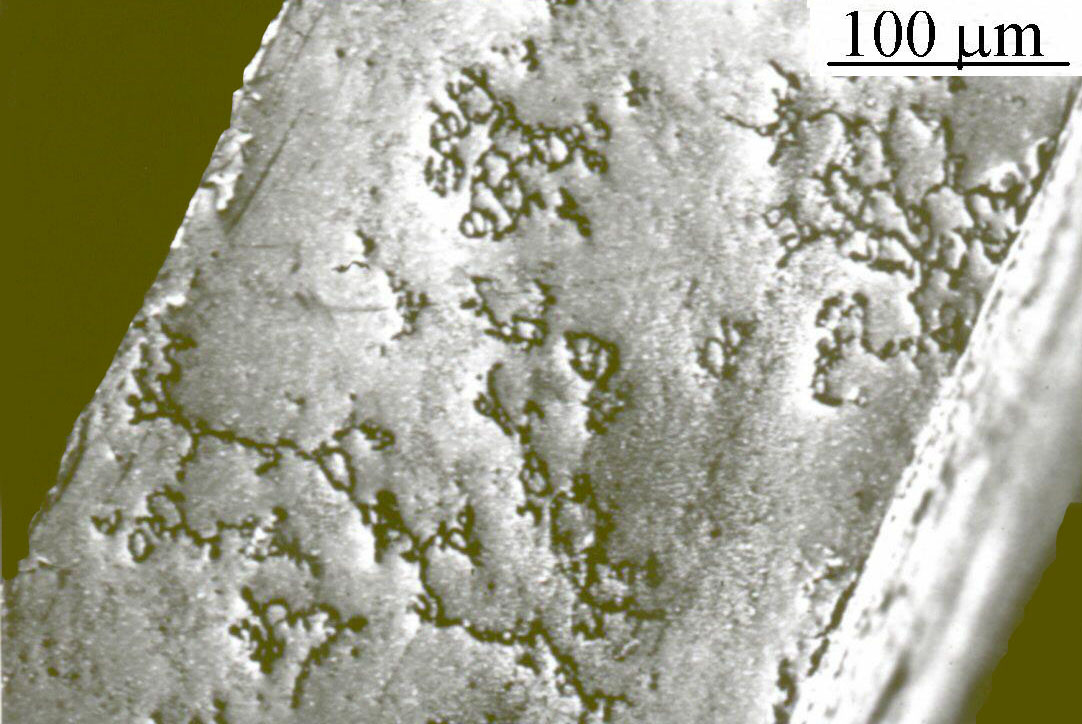
Intergranular cracks in the Pd-Ag cathode.

To overcome embrittlement and stress corrosion cracking by hydrogen and isotope, palladium cathode has to be alloyed, and the electrolytic process operates under conditions where phase transition or temperature critical does not occur. Another harmful phenomenon to control is to check uniformity in thickness or homogeneity of alloy after rolling, annealing or brazing, responsible for high mobility of the alloyed metal atoms. Numerous adequate elements including gold, nickel, silver or yttrium can be added to palladium to form solid solutions and to enhance the structural stability of the Pd-based membrane. Unfortunately, gold or nickel in the Pd alloy segregates on the surface owing to their high mobility, causing a reliability problem [18,19]. The mechanical stability is obtained for 25% weight of silver in the Pd-Ag alloy. At this content, the size of Pd-Ag lattice is expanded by the silver atoms before hydrogen charging. Hydrogen taking place has more space and distend less the lattice. Hydrogen will be less compressed when alloy is fully charged. As a result, stress corrosion cracking and mechanical modification of cathode are reduced. The transition temperature for the α ↔ β phases is 180 ${}^{{}^{\circ}}$C and this is widely lower in regard to pure palladium, encouraging the single phase formation [20]. In this system, if a suitable cathodic overpotential exists, hydrogen permeation takes place. In the case of no respect of critical temperature or critical overpotential of −450 mV referred to the normal hydrogen electrode (NHE), very high fictitious pressure is more and more progressing in the inlet surface leading to intergranular cracks (Figure 2) limiting the lifetime of palladium [11].

This phenomenon generates a significant force, which the Pd-Ag cathode has to sustain. The mechanism works as an electrolytic compressor for pure hydrogen at high pressure and for its storage. The relation between the differential fictitious pressure (Δ **P**) and the concentration gradient in the cathode is (Equation (5)):

(5) $$\Delta P=10^{1.5}\Delta C$$

Under misjudged conditions, two solid α- and β-phases coexist. In the palladium at lower content of silver, the three phases are present showing that the lattice is not expanded enough and hydrogen atoms are compressed in the inlet side leading to important local constraints into surface where the hydride concentration is high [11]. Thereby, it is obvious to introduce adequate amount of silver in palladium. However, for more than 25 wt % of silver, if mechanical constraints decrease due to the expanded lattice, permeability decreases by the fact to have removed away neighborhood sites used for diffusion. In the charging-diffusion concept, the temperature is decreasing hydride solubility and increasing permeability (Equations (2)–(4) and [1]). These two effects acting in contrast affect stress and locally can distort the palladium alloy if diffusion or solubility are not homogeneous like in the case of lateral diffusion at the interface of electrolyte-gas. As explained before, high fictitious hydrogen pressure is not entirely in advantage, and it can lead to the coexistence of three solid phases in the thickness of alloyed palladium if the conditions are not respected. The presence of three phases modifies the mechanical behavior and the stress corrosion cracking for cathode of palladium alloy [21,22]. The hydrogen atoms in the β-phase push away the palladium atoms further to take up their location in the face-centered cubic lattice. Consequently, hydrogen jumps for diffusion will decrease when the palladium atoms are too distant. This mechanism is a source of stress in the lattice, thickening, irreversible loss of permeability and over concentration of proton and hydrogen in the inlet side of cathode. Now, considering the part of immersed cathode shaped as a finger, a large amount of hydrogen or isotope is absorbed as a hydride phase in the inlet side. In the part above the surface of electrolyte, there is no hydrogen charging in the Pd-Ag cathode. Therefore, in the interface between these two parts, located at the surface of electrolyte, drastic structural changes lead to grain coarsening, loss of cohesion and mechanical constraints. The structural changes are also remarked on at the interface brazed where the palladium finger is connected to the tank for hydrogen storage [11]. In all cases, structural changes contribute to significant corrosion assisted cracking along the entire circumference interface. These prove existence of destructive phase in these zones. Similarly, repeated cycles of hydride loading and unloading by stopping heating or electrolysis for maintenance could also alter the phase transition and structure of Pd-Ag. These changes lead to the creation of lattice distortions and progressive irreversible mechanical strains and differential stress causing collapse of alloy [6]. When electrolysis is stopped without dehydrogenation, the outlet surface becomes β-phase, while the inlet surface becomes α-phase. An increase of approximately 10% in the value of mechanical hardness through the Pd-Ag cathode has been remarked. This is correlated with the stress attributable to deformation and creases. In this case, the cathode is not preserved of cracks [11]. To minimize this occurrence, removing hydrogen prior to shutdown is recommended. Draining hydrogen on the outlet side is accomplished by the use of vacuum, and removal hydrogen on the inlet side is realized by a flow of inert gas. Another common re-activating procedure is purging by heating or by electrochemical oxidation. Electrolytic oxidation acts in two ways: it eliminates the absorbed hydrogen as well as impurities deposited on the surface of the cathode during electrolysis. Once hydrogen is evacuated, the electrolyzer can be cooled down [11]. Therefore, this system suffers from a decrease in performance, showing that the cathode needs to be supported, and critical parameters have to be applied [11].

Although brittling by hydrogen appears to be well identified, there is also the decay helium. Desorption of ${}^{3}$He${}^{+}$ from palladium alloy cathode is very low, therefore its trapping by aging and the resulting cracking have to be minimized before its formation by evacuating tritium on stopping electrolysis as explained by the re-activating procedure.

## 3. Analysis of Model, Discussions and Solutions

**Figure 3:**
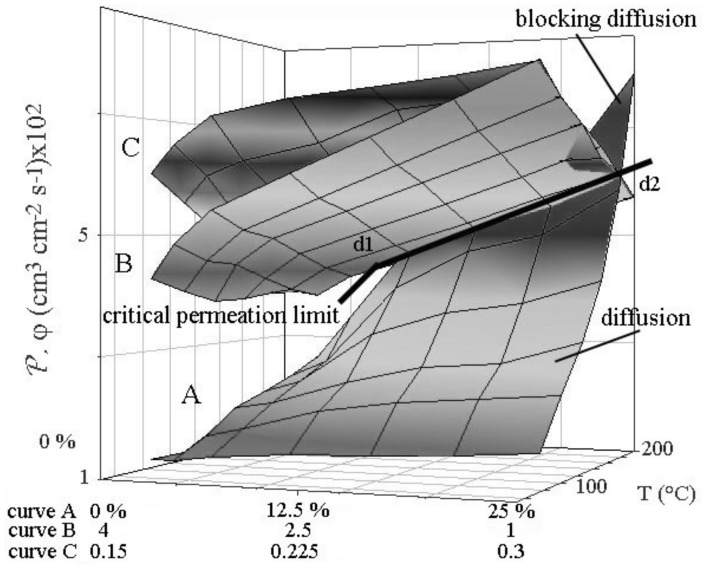
Permeability and flux. Effects of temperature and: Ag wt % (curve A), support thickness l mm for r: 0.25 μm (curve B), support porosity ($\varepsilon r{Ø}^{-1}$) μm for l: 1 mm (curve C), line d: limit of critical permeation.

Under these conditions, lattice expanded and Vickers hardness did not come back at the initial values (880 MPa) when all operating conditions are not respected. Depending on, cathode exhibits permanent and severe expansions, contractions as elongation and shortening, thickening, deformations, and irreversible loss of performance when it is not supported by porous support [11]. The knowledge of these parameters makes it possible to improve the mechanical characteristics of the cathode. To overcome deformation, porous support has to possess similar thermal expansion to palladium alloy to ensure good adhesion [22]. A dimensional failing ultimately leads to stress corrosion cracking then finger or disk failure. Validating the support by numerical simulation was made for Pd-Ag cathode. The data compiled from the Poiseuille and Knudsen’s equations show that the hydrogen flow (φ) is balanced for tortuous (τ) macropore radius (*r*) more than 0.5 μm, for volume porosity (ε) more than 0.4 and a thickness (*l*) less than 1 mm (Figure 3).

Equations can be summarized by the relative contribution of Equation (6) and of the viscosity of gas going through porosities:

(6) $$\varphi =\left[\frac{r\varepsilon \Delta P}{l\tau T^{0.5}}\right]$$

**Figure 4:**
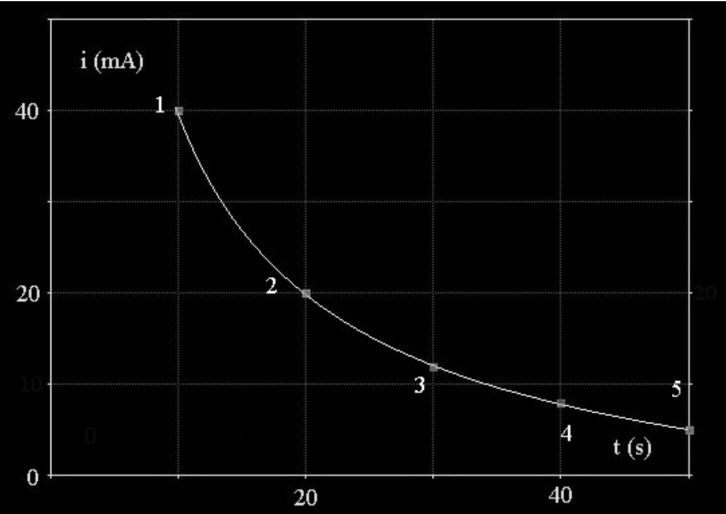
Current charging for hydrogen adsorption at the time of back diffusion.

**Figure 5:**
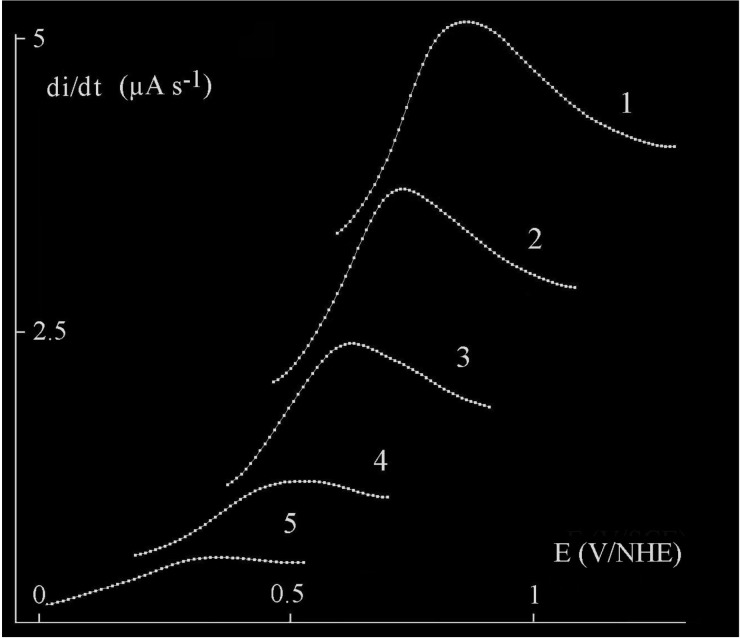
Derivative of anodic scans according to voltammograms. Scan number indicates peak of transient palladium complex lies to the adsorbed hydrogen current in Figure 4.

In this expression, **T** and Δ**P** are the absolute temperature and the differential pressure in the porous support [17]. Small porosities favor enrichment of the lightest gaseous isotope in the hydrogen storage tank. Simulation shows that beyond a critical value of permeation through the cathode, flow in the porous support limits the process for values higher than those of the critical permeation limit line of the figure. In this case, there is no more driving force for permeation, and interfacial pressure formation leads to inflating the cathode. Flow and permeation have to be equilibrated according Equations (2) and (6). As previously indicated, for safety reasons, the Pd-Ag cathode has to tolerate expansion in length, contraction with corrugation in width, thickening without any cracks or rupture to keep efficiency and durability at the selected temperature over the critical temperature. In this sense, connecting the open end of finger to the tank for hydrogen storage permits to minimize stress. A flat Pd-Ag cathode should be more stressed than the hollow finger. Required configuration depends also of the cold rolling and annealing procedures optimizing physical properties prior to permeation process. For this diffusion cathode technology, parameters required are 100 μm thick and 25 wt % of silver in alloy. All the previous inconveniences will vanish at a higher temperature than the critical temperature using concentrated NaOH or KOH+LiOH alkali electrolyte or pressurized water at 10${}^{2}$ kPa at low NaOH concentration in the electrolyzer made of high-grade alloy or stainless steel designed to be corrosion-resistant [7]. Water maintained higher temperature than the critical temperature and under high pressure allows it to remain in liquid form. At a high concentration of alkali metal, cathodic corrosion can be to occur on the cathode [23]. Cathodic corrosion is evidenced by the formation of transient complexes of palladium. Fast scanning rate cyclic voltammetry coupled to a high resolution digital oscilloscope is a very sensitive appropriate method for highlighting transient formation. Examining the derivative of the voltammograms after hydrogen charging and during back-diffused hydrogen (Figure 4), it is obvious to see a peak of palladium transient for different amounts of deposited hydrogen expressed as a current during back-diffusion (Figure 5). Oxydation for back-diffused hydrogen and current of transitory hydrogen complex are competitive on the direct voltammograms therefore transients are difficult to observe on these. On the derivative curves, the size and position of peak corresponding to rearrangement of the micro-structure, depend respectively on the amount of transients and adsorbed back-diffused hydrogen on the surface of the palladium. According to [24] , formation of transient palladium complexes formulated by [Na${}_{n}$PdH${}_{n}$] lies between 0 and −0.5 V versus RHE.

When back-diffused hydrogen occupies many superficial sites, it delays transformation of transients, and the derivative peak is more pronounced; therefore, transients are more important in number. In the absence of hydrogen, there is no more transient. Cathodic corrosion will therefore occur at a sufficiently negative potential to be in presence of adsorbed hydrogen promoting transient palladium formation. Cathodic corrosion would imply consequently a strong dependence of the concentration of adsorbed hydrogen and alkaline pH. Cathodic corrosion would weaken the superficial structure of palladium, leading to degradation of surface. Corrosion would concern hydride formulated by H${}^{-}$ and anionic nanoparticles represented by [PdH${}_{n}$]${}^{n-}$ where the main isolated is [PdH${}_{2}$]${}^{2-}$, justifying the low current on transient peak [25]. As a remark, cathodic corrosion and stress corrosion cracking act in concert since they form in the same range of potential in presence of hydrogen.

However, rather than using these conceivable parameters, high NaOH concentration or high pressure, posing important safety problem, this study was oriented towards more advanced alloy developed in the last part. The cathode design must overcome entirely these mechanical constraints and damage by stress corrosion cracking in order to work in safe operating conditions. Constraints are responsible for microscopic and macroscopic embrittlement and cracking of Pd-Ag cathode and actually this inconvenient is not really resolved, and the operating range is narrow for smooth operation. Inconvenient can be avoided using the palladium alloy with 9 wt % yttrium [26,27]. Rare earths or yttrium alloys suppress the α- and β-phases transition sensitive to embrittlement for temperature higher than 60 ${}^{{}^{\circ}}$C [28,29,30,31,32,33]. The Pd-Y alloy is considerably harder and has better mechanical stability, and no significant change is produced in the crystalline lattice by presence of hydride.

**Figure 6:**
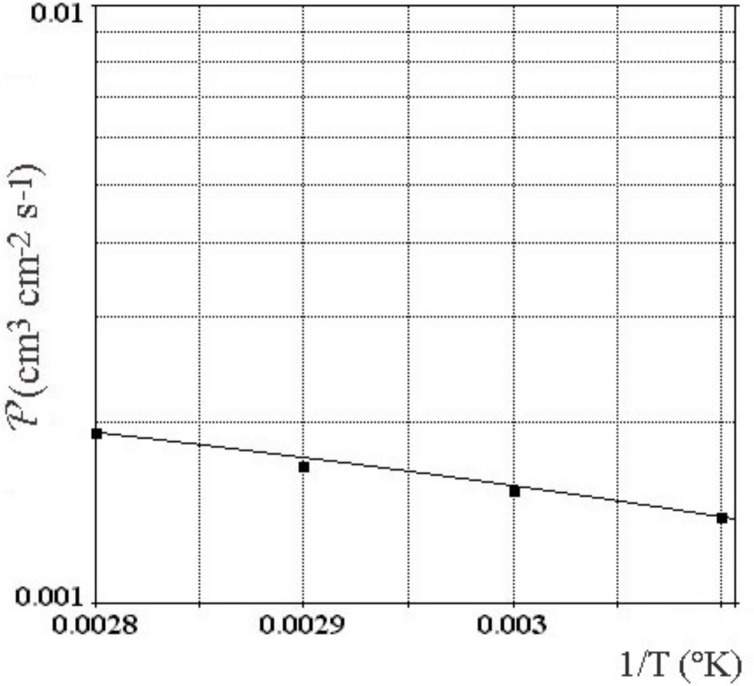
Effect of temperature for permeability in 9 wt % Y-Pd cathode membrane for hydrogen.

The Pd-Y alloy is annealed and hot-rolled by several step to reduce its thickness up to 80 μm with intermediate heating at 900 ${}^{{}^{\circ}}$C [34]. Successive annealing operation mitigates the strain hardening. Taking to account the annealed state, Vickers hardness is 2350 MPa. High hardness may lead to difficulties for shaping. This is why successive rolling and annealing were operated. Low elasticity is the predominant reason that finger is not launched on the market. Advantageously, the mechanical strength makes it possible to omit a porous flat support. As the critical temperature for formation of hydride and of phase transition vanishes at higher temperature than 60 ${}^{{}^{\circ}}$C, alloy is stable against embrittlement at a slightly higher temperature which permits a gain in permeation value. The temperature selected for electrolytic permeation is 80 ${}^{{}^{\circ}}$C, current is 80 mA cm${}^{-2}$ and NaOH concentration is 2 mol dm${}^{-3}$. Cathode surface is 100 cm${}^{2}$ for the industrial electrolyzer. To avoid the risk of forming an explosive gas mixture, the electrolyzer is swept with neutral gas during electrolyze. Moreover, of a practical interest, the Pd-Y alloy guarantees greater capacity for diffusion (Figure 6) with a factor more than 2–3 times, and lower capacity for cracking in comparison with Pd-Ag alloy at 25 wt % of silver [33]. The higher permeability has been attributed to a significantly enhanced solubility gradient, not by the diffusion coefficient [34]. As matter of fact, yttrium alloyed palladium increases performance for permeability, for the mechanical hardness and for low expansion. It will show a large durability without deterioration and a complete selectivity for hydrogen and isotope in time. Consequently the palladium-yttrium alloys will represent a considerable advance in the innovating technology of diffusion cathode for production of hydrogen and isotope. It will be a keystone for the selection of palladium alloy for this technology [7].

## 4. Conclusions

Considering the no-alloyed palladium, cracking and cathodic corrosion involve all surfaces subjected to charging hydrogen or tritium, while for the palladium-silver alloy, cracking is possible if content in silver, critical temperature or overpotential are not respected. Presence of adsorbed transient palladium complex confirms the cathodic corrosion by concentrated alkaline electrolyte in the region of hydrogen adsorption. Deformations of diffusion cathode are due to local modification of size in the lattice and presence of two hydride phases and a transition phase in palladium thickness. The alloy of palladium-silver is less sensitive to embrittlement than no-alloyed palladium. In the case of no-respect of critical parameters, the process will doom to a setback. Such cathode requires a porous support to overcome severe expansion, contraction, thickening and deformation in order to maintain its durability. Solution is also the palladium-yttrium alloy. It permits more easily to disregard these drawbacks encountered for the palladium and palladium-silver alloy used for the diffusion cathode.
